# Bony Lesion Severity in First-Time Posterior Shoulder Dislocation: The Role of Glenoid and Acromial Morphology

**DOI:** 10.1177/23259671261419373

**Published:** 2026-03-27

**Authors:** Malik Jessen, Lukas Willinger, Lucca Lacheta, Markus Schwarz, Chlodwig Kirchhoff, Sebastian Siebenlist, Peter Biberthaler, Philipp Zehnder

**Affiliations:** * Department of Sports Orthopaedics, TUM University Hospital Rechts der Isar, Technical University of Munich, Munich, Germany; † Department of Trauma Surgery, TUM University Hospital Rechts der Isar, Technical University of Munich, Munich, Germany; Investigation performed at TUM University Hospital Rechts der Isar, Technical University of Munich, Munich, Germany

**Keywords:** glenoid bone loss, scapular morphology, glenoid offset, acromial position

## Abstract

**Background:**

The influence of acromial morphology on the severity of glenoid bone loss (GBL) and reverse Hill-Sachs lesions (rHSL) in patients with first-time traumatic posterior shoulder dislocations (PSD) remains unclear.

**Purpose/Hypothesis:**

The purpose of this study was to assess the relationship between glenoid and acromial morphology and the severity of bone lesions, posterior GBL, and rHSL, in patients with first-time posterior PSD. It was hypothesized that specific morphological parameters, including glenoid offset and depth, as well as posterior acromial height, correlate with the extent of GBL and rHSL.

**Study Design:**

Cross-sectional study; Level of evidence, 3.

**Methods:**

In this monocentric, retrospective study, 24 patients with first-time PSD treated at a level 1 trauma center between 2011 and 2020 were analyzed. Scapular and humeral morphology was assessed using computed tomography and magnetic resonance imaging. Key parameters included measurements of glenoid and humeral morphology and acromial position. The correlation between anatomic measurements was evaluated using Pearson and Spearman correlation coefficients. Statistical significance was set at *P* < .05.

**Results:**

A GBL was present in 66.7% of patients with 20.8% showing a GBL >10%, while 95.8% demonstrated an rHSL. Smaller glenoid offset correlated with a deeper rHSL (*r* = −0.455; *P* = .03). Higher acromial position (posterior acromial height) correlated with larger GBL (*r* = 0.611; *P* = .01). A deeper glenoid correlated with a larger rHSL surface area (*r* = 0.624; *P* = .001). Reduced total acromial coverage showed a moderate negative correlation with GBL (*r* = −0.520; *P* = .04), while posterior acromial coverage did not correlate significantly with GBL.

**Conclusion:**

A posterior GBL and an rHSL can often be detected after an initial traumatic PSD. A higher positioned acromion and reduced total acromial coverage were found to correlate with larger posterior GBL, while a deeper glenoid and smaller glenoid offset correlated with larger and deeper rHSL.

Traumatic posterior shoulder dislocations (PSDs) are relatively rare, accounting for approximately 10% of initial shoulder dislocations,^34^ and are frequently caused by trauma or seizures.

They are often overlooked in initial diagnostics compared with anterior dislocations because of their more subtle clinical presentation.^22^ Standard radiographs can miss the diagnosis, making additional imaging techniques such as computed tomography (CT) or magnetic resonance imaging (MRI) crucial for accurate assessment.

PSD is often accompanied by glenoid and/or humeral bony injuries. The prevalence of posterior glenoid bone loss (GBL) is reported to be 69% to 86%,^11,16,40^ whereas the prevalence of a reverse Hill-Sachs lesion (rHSL) ranges from 22% to 86%.^1,27,35,36,42^ Similar to anterior GBL, attempts have been made to define a “critical” posterior GBL. Nacca et al^33^ demonstrated in a cadaveric model that posterior labral reconstruction failed to restore joint stability when posterior GBL exceeded 20%, indicating insufficient biomechanical results beyond this threshold. Recent clinical evidence suggests that considerably smaller GBLs have significant consequences. Arner et al^4^ investigated the association between posterior GBL and failure rates following posterior labral repair outcomes. Patients with failed labral repair demonstrated a mean GBL of 6.8%, compared with 4.6% in those with successful outcomes. Notably, a GBL of 11% was linked to a 10-fold increase in failure risk, while a 15% GBL was already associated with a 24-fold increase. Wolfe et al^40^ similarly reported a 44% failure rate for arthroscopic repair when GBL exceeded 14%. These findings highlight the relevance of posterior GBL as a predictor of labral repair failure. Although definitive thresholds remain to be established, current evidence suggests that clinically relevant posterior GBL may occur at levels below 10%.^21^

Furthermore, an elevated retroversion has been suggested to be a potential risk factor for PSD.^34^ However, posterior glenoid osteotomy to correct increased retroversion leads to varying results.^17^ Hence, acromial morphology has also gained attention as a contributing factor to posterior instability. Some studies have investigated the influence of the anatomic position of the acromion as another possible risk factor for both posterior shoulder instability^7,19,28,29^ and a posterior GBL.^25^ It has been hypothesized that the acromion is positioned higher and appears flatter in patients with PSD than in patients with posterior shoulder instability.^18,19,25,28^ This altered morphology may reduce posterior containment of the humeral head, thereby increasing the likelihood of instability and bony injury. The reduced posterior coverage of the humeral head potentially diminishes the effectiveness of a bony posterior stabilizer against a posteriorly directed force on the humeral head.^19^ However, the clinical presence and significance of this observation remain unclear.

Understanding the interaction between these anatomic factors is crucial for identifying patients who may be at risk for more severe osseous injury following first-time PSD. While the extent to which individual morphologic variations contribute to treatment outcomes remains unclear, identifying correlations between glenoid morphology, acromial position, and rHSL severity may improve risk stratification for potential treatment regimes.

This study aimed to describe acromial, glenoid, and humeral parameters in patients with first-time traumatic PSD to assess their relationship to the severity of posterior GBL and rHSL. It was hypothesized that specific morphological factors, including glenoid offset and depth and posterior acromial height (PAH), correlate with the extent of GBL and rHSL.

## Methods

### Study Population

The study was approved by the ethics committee of the medical faculty of the Technical University of Munich, Germany, and was conducted according to the Declaration of Helsinki and its amendments.

A monocentric, retrospective case study analyzing patients with a first-time traumatic PSD (type A2 according to the ABC classification of posterior instability^32^) who were treated in a level 1 trauma center emergency department was performed between 2011 and 2020. An electronic documentation system was used to obtain the demographic data and injury mechanism. Patients with PSD and complete CT or MRI imaging of the shoulder (in particular, the entire scapula had to be depicted) were included. A comparability of measurements between CT and MRI has already been investigated in previous studies.^9,13,26,38^ Only the first CT examination was evaluated for patients who underwent multiple advanced imaging, including CT and MRI scans. The inclusion criterion was a first-time traumatic PSD. Exclusion criteria were defined as follows: patients without complete visualization of the scapula in CT or MRI scan; patients with anterior or inferior shoulder dislocation; accompanying humeral head fracture; time between initial PSD and CT/MRI imaging exceeding 4 weeks, as secondary bone remodeling and resorption could alter the accuracy of osseous defect measurements; patients with a history of inflammatory arthropathy; patients with prior trauma (shoulder dislocation or fracture) to the ipsilateral shoulder girdle including the scapula, clavicle, or acromion; and patients with a history of prior ipsilateral shoulder instability.

### Image Measurements

All CT scans were performed in the institution’s radiology department. CT scans were obtained by a 64-slice CT scanner (Somatom Sensation 64; Siemens) with a slice thickness of 1 mm. MRI was used for patients without an available CT scan. All radiographic parameters were assessed using a picture archiving and communication system workstation certified for clinical use (IDS7 21.2; Sectra).

A standardized imaging plane using multiplanar reconstruction was applied for both the axial and the sagittal planes for all measurements in CT and MRI scans obtained. A standardized axial imaging plane was used, according to Moroder et al.^30^ Standardized sagittal imaging plane, especially for a glenoid en face view, was created as described by Griffith et al.^12^ The images of left shoulders were reversed so that all images appeared as right shoulders, ensuring that the 9 o’clock position corresponded to the posterior glenoid in all cases.

### Scapular Measurements

**Figure 1.**
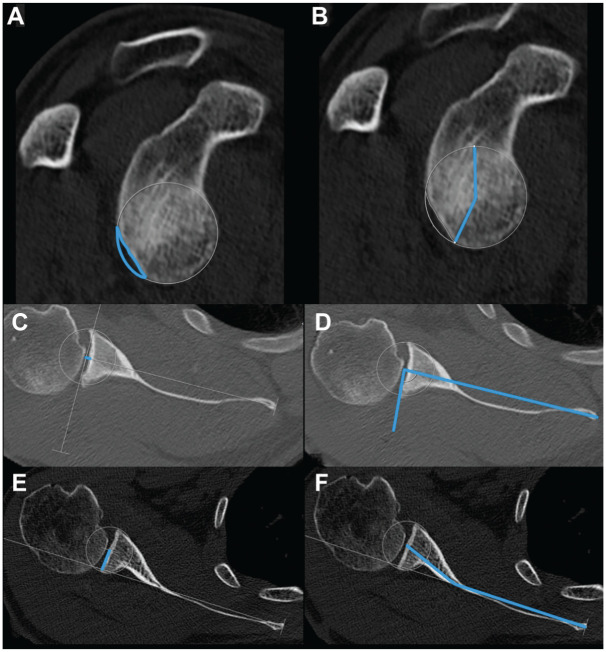
Glenoid measurements in the (A-B) sagittal and (C-G) axial planes based on 2-dimensional computed tomography multiplanar reconstruction. (A) Glenoid bone loss (GBL) is measured using the surface area ratio, calculated as the ratio between the manually segmented defect area and the total glenoid surface area defined by the best-fit circle. (B) Angular measurement to determine the clockface position for the caudal boundary of the GBL. (C) Glenoid depth, (D) glenoid retroversion, (E) glenoid offset, and (F) neck angle. Blue lines illustrate the performed measurements.

The glenoid axis was defined as the tangent between the anterior and posterior rims of the glenoid. The glenoid depth was measured from the glenoid axis to the deepest point of the glenoid. The glenoid version was measured using Friedman et al’s^10^ method. The neck angle and glenoid offset were utilized to characterize the orientation and spatial position of the glenoid vault relative to the scapular blade. Both parameters were measured according to Akgün et al,^2^ and the scapular blade axis was used as described by Hoenecke et al.^20^ Representative measurements are shown in Figure 1 (A and B).

To measure the GBL, a best-fit circle was first placed on the glenoid en face view, as previously described.^14,39^ GBL was quantified using the best-fit circle method and the surface ratio (Figure 1).

The location of the GBL was described using the clockface position method by Livesey et al.^26^ Three different locations were measured using the clockface convention: the time of the superior and inferior boundaries of theGBL and the time at the location of the maximal GBL diameter. Measurements are represented in Figure 1 (C-F).

**Figure 2.**
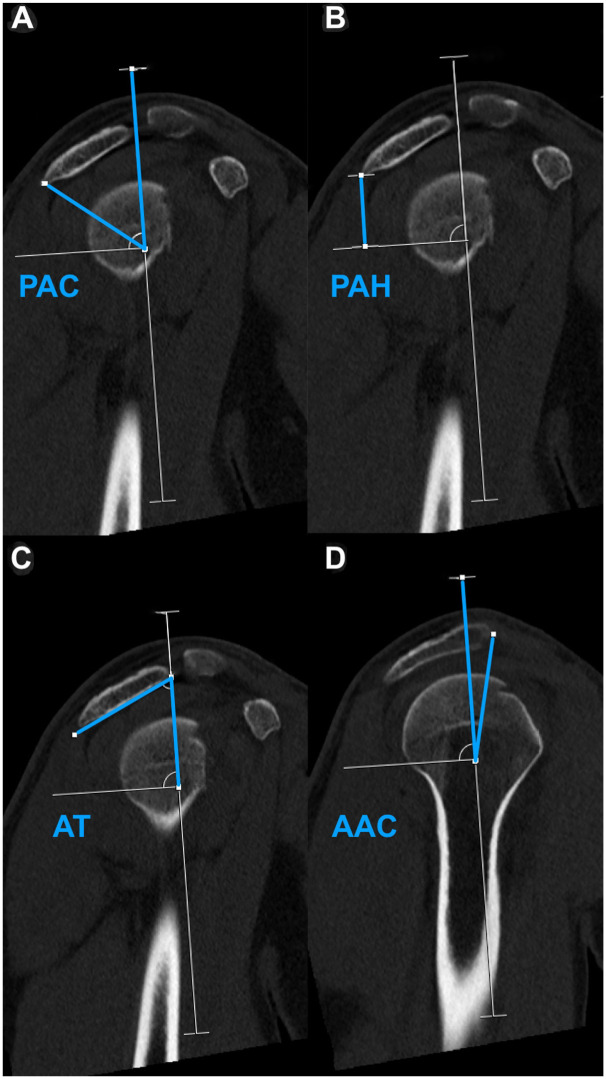
Acromial measurements in the sagittal plane based on 2-dimensional computed tomography multiplanar reconstruction. Blue lines illustrate the performed measurements. (A) Posterior acromial coverage (PAC). (B) Posterior acromial height (PAH). (C) Acromial tilt (AT). (D) Anterior acromial coverage (AAC).

Subsequently, the acromion was measured in a sagittal imaging plane using the 2-dimensional CT multiplanar reconstruction, and measurements were adapted to previously described methods^3,6,8,25,28^; a scapular reference line was first drawn from the inferior scapular angle through the center of the glenoid, represented by the best-fit circle. Based on previously described methods by Meyer et al,^28^ Arner et al,^3^ and Livesey et al,^25^ the following parameters were obtained (Figure 2): acromial tilt (AT; the angle between the scapular reference line and the inferior edge of the acromion), PAH (the perpendicular distance from the scapular reference line to the most posterior point of the inferior acromion), anterior acromial coverage (AAC; the angle between the anterior boundary of the acromion and the scapular reference line; the AAC angle is assigned a negative value if the acromion does not cross the reference line and a positive value if it crosses the line), posterior acromial coverage (PAC; the angle between the scapular reference line and the posterior boundary of the acromion), and the total acromial coverage (TAC; calculated as the sum of AAC and PAC).

### Humeral Measurements

Using the axial imaging plane, a best-fit circle was first determined on the humeral head to identify its center and surface area. The location of the rHSL was then determined using clockface positions as described by Yang et al.^42^ The bicipital groove was defined as the 12 o’clock position. Two clockface positions were determined: anterior and medial boundaries of the rHSL.

**Figure 3.**
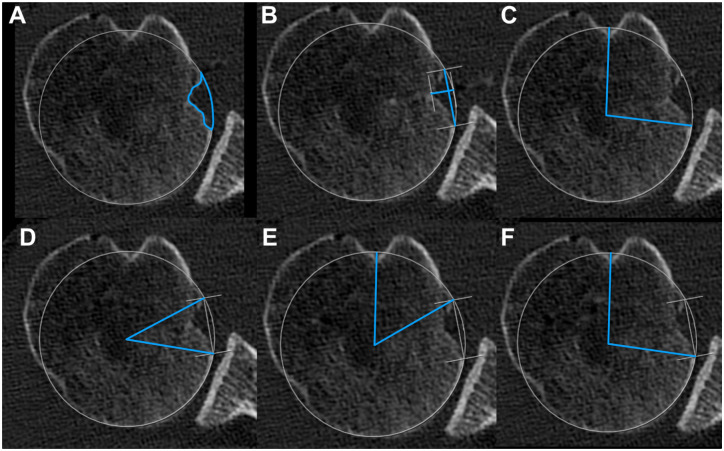
Measurements of the reverse Hill-Sachs lesion (rHSL) in the axial plane based on 2-dimensional computed tomography multiplanar reconstruction. Blue lines illustrate the performed measurements. **(**A) Osseous defect of the rHSL. (B) Width and maximal depth of rHSL. (C) Angular measurement to determine the clockface position for the posterior boundary of the rHSL. (D) Alpha angle, (E) beta angle, and (F) gamma angle.

The size of the rHSL was determined by measuring its width and maximal depth. To assess the osseous defect, the area of the rHSL was related to the area of the humeral head. The area of the rHSL was manually outlined within the best-fit circle, similar to the defect area of the GBL. Finally, the alpha, beta, and gamma angles were determined as previously described by Moroder et al.^31^ Measurements are represented in Figure 3.

**Figure 4.**
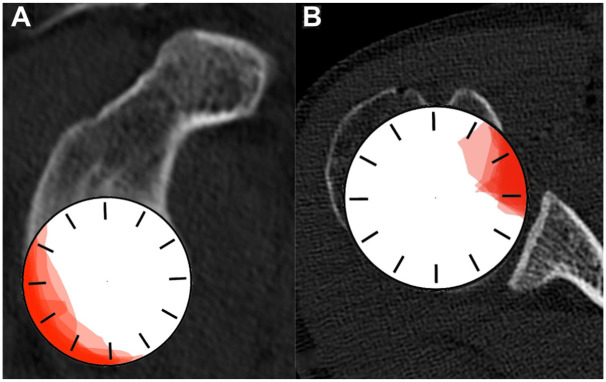
Clockface position of (A) the glenoid bone loss (GBL) and (B) the reverse Hill-Sachs lesion (rHSL). A clockface with 12-hour markings is depicted in white, while the (A) GBL and (B) rHSL of all shoulders are overlaid in red for visualization.

Graphical visualization of GBL and rHSL in clockface position (Figure 4) was created using Pixelmator Pro (Version 3.6.16; Pixelmator Team Ltd.).

### Statistical Analysis

Two independent raters, both orthopaedic sports medicine residents (M.J. and P.Z.), performed all measurements at different time points. The intraclass correlation coefficient (ICC) with the 95% CI was calculated for all measurements. The agreement was graded as recommended by Landis and Koch^24^ (ICC: 0.00-0.20, slight agreement; 0.21-0.40, fair agreement; 0.41-0.60, moderate agreement; 0.61-0.80, substantial agreement; 0.81-1.00, strong agreement). The results were given as the mean, standard deviation, range, or number and percentage. The Kolmogorov-Smirnov test was used to test for normal distribution. Correlation analyses were performed using the Pearson correlation coefficient (*r*) for normally distributed data, and the Spearman correlation coefficient (ρ) was used for nonnormally distributed data. The strength of correlation was categorized as follows: 0.00-0.10, negligible correlation; 0.10-0.39, weak correlation; 0.40-0.69, moderate correlation; 0.70-0.89, strong correlation; and 0.90-1.00, very strong correlation.^37^ The results were given as the mean and standard deviation or as the number and percentage. Two-tailed *P* values were calculated with a significance level set at *P* < .05. Data analysis was performed with Prism software (Version 10.0.3; GraphPad) and SPSS Statistics (Version 30.0.0.0; IBM Corp).

## Results

Overall, 24 patients (24 shoulders) were enrolled in this retrospective study. A total of 21 were male and 3 were female, with a mean age of 42.3 years (range, 19-75 years). The injury mechanisms were as follows: bicycle fall (9 patients), skiing accident (3 patients), seizure (3 patients), fall from a low height of <2 m (3 patients), sports injuries (2 patients), motorcycle accident (2 patients), and car accident (2 patients). For 21 out of 24 shoulders, CT scan data were used for measurements, and for 3 shoulders, MRI scan data were used. The CT slice thickness was 1 mm in all cases. MRI slice thickness ranged from 1 to 3 mm. All measurements showed strong agreement between the 2 raters, except for the clock position of the GBL (substantial agreement). ICCs are summarized in Table 1. The results of the radiological measurements of the glenoid, acromion, and rHSL are summarized in Table 2. A GBL was present in 16 patients (66.7%), of whom 5 patients had a GBL of >10%. An rHSL was present in 23 patients (95.8%).

**Table 1:** Calculated ICCs for All Measurements*^*a*^*

			95% CI		
		ICC	Lower bound	Upper bound	Agreement
Glenoid	Glenoid depth	0.890	0.744	0.952	Strong
	Glenoid version	0.882	0.726	0.949	Strong
	Neck angle	0.895	0.719	0.957	Strong
	Glenoid offset	0.913	0.801	0.962	Strong
	GBL				
	Best-fit circle	0.933	0.811	0.976	Strong
	Surface	0.968	0.910	0.989	Strong
	clockface position for GBL				
	Superior boundary	0.879	0.634	0.960	Strong
	Inferior boundary	0.688	0.122	0.891	Substantial
	Maximal diameter	0.686	0.096	0.890	Substantial
Acromion	Acromial tilt	0.980	0.954	0.991	Strong
	Posterior acromial height	0.976	0.944	0.989	Strong
	Anterior acromial coverage	0.963	0.915	0.984	Strong
	Posterior acromial coverage	0.982	0.959	0.992	Strong
	Total acromial coverage	0.953	0.890	0.980	Strong
	Clockface position for rHSL				
	Anterior boundary	0.818	0.580	0.922	Strong
	Medial boundary	0.921	0.814	0.967	Strong
rHSL	rHSL width	0.934	0.812	0.974	Strong
	rHSL depth	0.971	0.930	0.988	Strong
	rHSL surface	0.991	0.978	0.996	Strong
	Alpha angle	0.848	0.647	0.935	Strong
	Beta angle	0.818	0.580	0.922	Strong
	Gamma angle	0.921	0.814	0.967	Strong

a GBL, glenoid bone loss; ICC, intraclass correlation coefficient; rHSL, reverse Hill-Sachs lesion.

**Table 2:** Radiological Measurements*^*a*^*

		Mean	SD	Range
Glenoid	Glenoid depth, mm	2.0	1.24	0-4.9
	Retroversion, deg	8.0	4.9	−5.8-15.5
	Neck angle, deg	167.1	4.7	158.2-176.5
	Glenoid offset, mm	7.8	3.2	1.4-13.4
	GBL (n = 16)			
	Best-fit circle, %	9.2	4.7	6.2-26.1
	Surface, %	6.8	3.7	3.0-18.6
	Clockface position for GBL			
	Superior boundary	09:25	0:42	09:18-10:24
	Inferior boundary	06:13	0:43	05:00-07:09
	Maximal diameter	08:07	1:01	06:21-10:29
Acromion	Acromial tilt, deg	65.6	8.6	53.1-81.8
	Posterior acromial height, mm	22.5	4.6	11.5-31.5
	Anterior acromial coverage, deg	4.9	6.4	−5.7-16.5
	Posterior acromial coverage, deg	58.7	7.6	41.3-73.2
	Total acromial coverage, deg	63.6	6.2	52.9-72.0
rHSL	clockface position for rHSL (n = 23)			
	Anterior boundary	01:51	0:21	01:08-02:34
	Medial boundary	03:02	0:17	02:27-03:35
	Width, mm	14.1	5.2	8.9-33.0
	Depth, mm	7.1	3.1	2.9-13.4
	Surface, %	4.5	3.4	0.9-16.0
	Alpha angle, deg	38.6	13.7	23.0-79.2
	Beta angle, deg	57.2	18.1	22.2-116,5
	Gamma angle, deg	91.2	8.7	73.6-107.7

a GBL, glenoid bone loss; rHSL, reverse Hill-Sachs lesion.

Further correlation analysis revealed significant relationships between specific anatomic parameters. A higher acromial position (PAH) was significantly associated with a larger GBL (moderate correlation; ρ = 0.611; *P* = .01). There is no significant correlation between GBL and PAC. Additionally, a smaller TAC had a moderate correlation with a larger GBL (ρ = −0.520; *P* = .04). A deeper glenoid was significantly associated with a larger rHSL surface area (moderate correlation; ρ = 0.624; *P* = .001), while a smaller glenoid offset was related to a deeper rHSL (moderate correlation; ρ = −0.455; *P* = .03). Figure 4 illustrates the clock positions of the GBL and rHSL.

## Discussion

The main findings of this study were a high acromial position associated with a higher posterior GBL and that a deeper glenoid and a smaller glenoid offset were associated with a larger rHSL. In addition, reduced TAC showed a negative correlation with the extent of GBL. This study offers new insights into the relationship between anatomic factors linked to first-time PSD. These factors are commonly considered indications for more extensive surgical treatment in first-time anterior shoulder dislocations, such as Remplissage or bone block transfer.^15,23^

While prior investigations have primarily focused on glenoid anatomy, our study highlights the importance of the acromion as a potential contributor to the size of posterior GBL and, therefore, potentially contributes to posterior instability.

In a retrospective study, Livesey et al^25^ demonstrated that patients without GBL had a steeper AT and greater PAC compared with those presenting with GBL, suggesting that a flatter acromion and reduced posterior coverage may be associated with the presence of GBL.

In the present study, no correlation was found between PAC and GBL or between AT and GBL. However, GBL correlated with PAH and TAC, indicating that both a higher positioned acromion and reduced overall acromial coverage were associated with a greater extent of GBL. Differences between the findings of Livesey et al^25^ and those of the present study may be attributed to variations in the patient populations. In the study by Livesey et al,^25^ all patients with posterior shoulder instability were included, comprising both acute cases and those without a documented dislocation or subluxation but with a positive Jerk or Kim test on clinical examination. In that cohort, acromial height did not differ between patients with and without GBL. In the present cohort, all patients sustained a first-time PSD, and interestingly, the extent of GBL correlated with PAH and TAC. Whether a higher positioned acromion also contributes to more pronounced (chronic) posterior shoulder instability or to recurrent instability should be investigated in future studies using clinical outcome data.

A potential explanation for greater GBL in a higher positioned acromion is that a higher acromion may reduce the posterior containment of the humeral head, leading to altered force distribution. A recent biomechanical study supports this theory. Hochreiter et al^19^ examined posterior shoulder stability with regard to acromial height and tilt in 8 fresh-frozen human cadaveric shoulders. A high and flat acromion leads biomechanically to reduced posterior shoulder stability. The authors concluded that the acromion is a mechanical buttress to posterior humeral head displacement. Therefore, PSD in the presence of a higher positioned and flattened acromion could be associated with a larger GBL. However, whether acromial morphology is a causal factor for a greater GBL remains unclear. The clinical implications of these findings warrant further investigation, particularly in the context of surgical decision-making and stabilization strategies for posterior shoulder instability. Future studies should explore whether anatomic variations such as increased acromial height or reduced coverage influence clinical outcomes following surgical treatment (eg, posterior labral reconstruction).

The radiological assessment revealed a high prevalence of GBL with a size exceeding 10% (20.8%) and rHSL (95.8%) (Table 2), emphasizing the frequent osseous involvement in first-time posterior dislocations. These findings support the notion that even initial posterior dislocations often implicate structural damage, which may have consequences for recurrent instability and surgical decision-making.

Regarding the location of the GBL using the clock position method, the results are comparable with previous descriptions of the GBL position.^26^ A posteroinferior location of the GBL is also observed in our present cohort (Figure 4). Livesey et al^26^ also reported a moderate positive correlation between the size of the posterior GBL and glenoid retroversion. Unlike Livesey et al,^26^ we did not find a correlation between glenoid retroversion and GBL, which is consistent with the findings of Beaulieu-Jones et al.^5^ One hypothesis could be that during a posterior dislocation, the humeral head does not exert increased force on the glenoid in cases of a more retroverted glenoid, thereby not causing greater damage than a neutrally verted glenoid.

The prevalence of rHSL in the present cohort (95.8%) was notably higher than the rates reported in previous studies, which ranged from 21.5% to 86%.^1,27,35,36,42^ The reasons for the varying prevalence are multifactorial. The inclusion criteria differed among the studies, and rHSLs were also detected using different assessment tools, including radiography, CT, or MRI. Moreover, this difference may, at least in part, be attributed to a selection bias inherent in our study population. All patients in the present cohort were emergency department cases who sustained an acute traumatic PSD, and most of them underwent immediate high-resolution CT scanning as part of their diagnostic workup.Consequently, these injuries may represent higher-energy trauma events, whereas patients with less severe mechanisms of injury may not routinely present to an emergency setting or undergo CT evaluation.

Yang et al^42^ reported a prevalence of 51% in their CT-based analysis of patients with traumatic (first-time and recurrent) PSD, which falls within the previously described spectrum. The higher prevalence observed in the present study may therefore be attributed to differences in inclusion criteria and imaging protocols. Specifically, Yang et al included both first-time and recurrent cases, whereas our cohort consisted exclusively of acute, first-time traumatic dislocations evaluated by high-resolution CT.

Consistent with Yang et al,^42^ the present results confirmed that the rHSL is typically located on the anteromedial aspect of the humeral head, with comparable clockface coordinates (approximately 1:30-3:00). The present data, together with those of Yang et al, represent the first detailed descriptions of the exact location of the rHSL. The mean depth of the rHSL in the present series (7.1 ± 3.1 mm) was slightly greater than that described by Yang et al (5.8 ± 2.2 mm), while the mean width was similar (14.1 ± 5.2 mm vs 11.1 ± 3.6 mm, respectively). This difference in depth may reflect the higher proportion of acute traumatic cases in our study, resulting in more pronounced impaction of the humeral head against the posterior glenoid rim.

Moreover, our findings indicate that a deeper glenoid is associated with a larger rHSL, suggesting that glenoid concavity may play a role in rHSL formation during posterior dislocation events. The inverse relationship between glenoid offset and rHSL depth may reflect changes in joint congruency, which could affect how the humeral head engages with the glenoid during posterior instability episodes.

In the presented cohort, a significant correlation was found between a deeper glenoid depth and a larger overall rHSL area. A correlation between a glenoid depth >2 mm and a larger HSL has previously been described for anterior shoulder dislocations.^41^ In the study by Wu et al, an HSL in the setting of a glenoid depth >2 mm also appeared to be more medialized compared with HSL in shallower glenoids, increasing the risk of an off-track HSL. The authors reported that patients with deeper glenoids experienced more dislocations, indicating a direct relationship between glenoid characteristics and HSL severity. To our knowledge, a correlation between glenoid depth and rHSL in PSDs has not been previously described.

Furthermore, the patient cohort presented showed a correlation between a deeper rHSL and a smaller glenoid offset. A reduced glenoid offset means that the glenoid lies closer to the plane of the scapula. A reduced glenoid offset may limit the posterior translation space of the humeral head during a PSD, leading to greater impaction against the posterior glenoid rim. This hypothesis could explain the observed association with a deeper rHSL but requires further biomechanical validation.

Our findings suggest that specific anatomic risk factors, such as a higher acromial position, smaller glenoid offset, and a deeper glenoid, are associated with a larger GBL and a larger rHSL, which may contribute to persistent posterior instability. Future research should focus on determining whether these morphological characteristics directly lead to recurrent instability and identifying potential threshold values that could serve as protective factors against recurrent dislocation.

### Limitations

This study has some limitations. Its retrospective design introduces a potential selection bias, as only patients treated in the emergency department were included, and documentation may be incomplete. Furthermore, the sample size of only 24 patients is relatively small. The observed correlations are suggestive of a relationship between acromial morphology and posterior instability, though larger studies are required to confirm this association. Nevertheless, the homogeneous inclusion of patients with acute, first-time PSD enhances the internal validity of the results, while limiting their generalizability to other (chronic) posterior instabilities. Another limitation is the use of external MRI data, which may vary in image quality. Although previous studies have demonstrated high comparability between CT and MRI for scapular and glenoid morphology, subtle measurement inaccuracies cannot be completely ruled out. The CT slice thickness was 1 mm, while the MRI slice thickness ranged between 1 and 3 mm. Despite this minor difference, image quality and standardization were sufficient to ensure consistent morphologic assessment. Despite standardized imaging techniques with strong ICC values, subtle measurement inaccuracies cannot be entirely ruled out.

Additionally, all left shoulder images were horizontally flipped to match right-sided orientation for standardization purposes. To our knowledge, this manipulation did not introduce any measurable error or artifact, but this cannot be entirely excluded. For the visualization and overlapping of GBL and rHSL of all shoulders, no artifacts or errors were known to us. This is because all GBL and rHSL were standardized and plotted using a clockface scheme, allowing precise and reproducible superimposition.

Future studies should validate these findings and integrate biomechanical analyses and clinical outcome studies to further elucidate the role of scapular morphology in posterior shoulder instability.

## Conclusion

A posterior GBL and an rHSL can often be detected after a first-time traumatic PSD. A higher positioned acromion and reduced acromial coverage were found to correlate with larger posterior GBL. Furthermore, a deeper glenoid and a smaller glenoid offset correlated with larger and deeper rHSL. These findings highlight morphological relationships that may contribute to understanding bony lesions in posterior shoulder instability.
